# Dietetics

**Published:** 1905-07-29

**Authors:** 


### DIETETICS.

(*Continued from page* 295.)

**Alcohol.**—In health a certain amount of alcohol is oxidised in the body, according to Chittenden,[18] but the facts that when large quantities are taken it is excreted unchanged, and that aldehydes and acetic acid products, showing an incomplete breaking up of foodstuffs, are formed indicate that it is not an advantageous food. Experiments as to the amount of alcohol oxidisable in dogs suggest that an average healthy man could oxidise 168 grams of pure whisky, or a quart of port wine. The rôle of alcohol when absorbed is not tissue-building but energy-producing. As regards the effect of alcohol on metabolism Beebe has shown that in healthy young men unaccustomed to the drug a noticeable increase in uric acid and purin bodies takes place in the urine. This effect is still greater with certain alcoholic drinks, such as port wine. Lusk[19] points out that Mandel's experiments show that this uric acid is of exogenous origin. A fasting man excreted the same amount of uric acid whether he took 900 cc. of whiskey or not. If the whiskey were taken with food the uric acid was increased. Chittenden holds that the absorbed alcohol reaches the liver, where some cleavage product is formed, bringing down some nuclein products. Lusk, however, does not think that any reduction of the oxidising power of the liver occurs, but admits that the alcohol may damage certain exogenous ferments. Woodruff[20] states that biological opinion inclines to the view that the pancreas, by means of a ferment, converts carbohydrates into alcohol, which is then oxidised in the tissues with the production of energy, and that fats are synthesised in the cells from alcohol and a fatty acid. In disease Chittenden states that the ingestion of alcohol is not followed by an increase in uric acid production. Peabody[21] sums up the uses of alcohol in disease as (1) externally as an antiseptic and as an application in neuritis, phlebitis, suppurative and phlegmonous conditions, when it acts by dilating the blood-vessels and relieving pressure. It should be applied early in the form of saturated compresses, and covered with gutta percha. It should not be used where the epidermis is very thin, as on the scrotum or in children, unless highly diluted. (2) Internally as an antidote in carbolic acid poisoning; as a cardiac stimulant, especially in cases of fever with much delirium, high temperature, and prostration. In the tropics Woodruff[22] considers that a moderate amount of alcohol is not only beneficial, but an actual necessity. From observations made on large numbers of soldiers of the United States army in the Philippines, he concludes that those taking excessive amounts of alcohol are less affected by the badness of the climate than total abstainers. He points out that in certain diseased conditions such as septicæmia, yellow fever, and cholera, large quantities of alcohol are beneficial and well tolerated. The exhaustion which results from residence in the tropics he considers requires similar treatment. He quotes experiments of Roos, who showed that guinea-pigs fed with alcohol were kept in better condition as regards resistance to disease, fecundity, longevity, &c., than control animals; and those of Mercoli, who showed that the blood of alcoholics had greater antitoxic power against tuberculin than that of abstainers. Those, however, whose tissues have been damaged by prolonged excess of alcohol show no antitoxic power in their blood. Woodruff points out that the first effect of a tropical climate is stimulating, and that exhaustion only occurs after prolonged residence. He thinks that the extra amount of water consumed by those taking alcohol may be a factor in the good effects produced. With most other authorities he recommends that the alcohol should be taken with the evening meal. He does not think that excessive use of alcohol is more deadly in the tropics than elsewhere; chronic alcoholics die sooner in the tropics than at home, but this is the case with all whose tissues have been weakened by age, disease, or any other cause.

[18] Med. Rec., March 4, 1905, p. 356. [19] Ibid., p. 357. [20] Ibid., Dec. 17, 1904, p. 961. [21] Ibid., March 4, 1905, p. 356. [22] Loc. cit.

